# Diuron exposure and Akt overexpression promote glioma formation through DNA hypomethylation

**DOI:** 10.1186/s13148-019-0759-1

**Published:** 2019-11-14

**Authors:** Joséphine Briand, Arulraj Nadaradjane, Gwenola Bougras-Cartron, Christophe Olivier, François M. Vallette, Pierre-François Cartron

**Affiliations:** 1 CRCINA, INSERM, Université de Nantes, Nantes, France; 2 LaBCT, Institut de Cancérologie de l’Ouest (ICO), Saint Herblain, France; 3 Cancéropôle Grand-Ouest, réseau Epigénétique (RepiCGO), Nantes, France; 4 EpiSAVMEN Network, Nantes, France; 5 Service de toxicologie, Faculté de pharmacie de Nantes, Nantes, France; 6 LabEx IGO “Immunotherapy, Graft, Oncology”, Nantes, France; 7 CRCINA, INSERM U1232, Equipe Apoptose & Progression Tumorale, LaBCT ICO Site R Gauducheau, Boulevard du Pr J Monod, 44805 Saint Herblain, France

**Keywords:** DNA methylation, Diuron, Gliomagenesis, Apoptosis, PD-L1

## Abstract

**Background:**

Diuron is an environmental component listed as a likely human carcinogen. Several other studies report that diuron can be oncogenic for bladder, urothelial, skin, and mammary cells. No study mentions the putative effect of diuron on the glioma occurrence.

**Objectives:**

We here wanted to investigate the effects of diuron exposure on the glioma occurrence while wishing to incriminate a putative implication of DNA methylation modulation in this process.

**Methods:**

In in vivo model of glioma, diuron exposure was firstly compared or combined with oncogenic overexpressions already known to promote gliomagenesis. ELISA quantifying the 5-methylcytosine level on DNA was performed to examine the global DNA methylation level. Quantitative real-time polymerase chain reaction and proximity ligation in situ assay were performed to identify the molecular causes of the diuron-induced changes of DNA methylation. The signatures diuron-induced changes of DNA methylation were analyzed in a cohort of 23 GBM patients.

**Results:**

Diuron exposure is not sufficient to promote glioma, such as the oncogenic overexpression of Akt or Ras. However, the combination of diuron exposure and Akt overexpression promotes glioma. We observed that the diuron/Akt-induced glioma is characterized by three phenotypic signatures characterizing cancer cells: a global DNA hypomethylation, a loss of sensitivity to cell death induction, and a gain of signals of immune escape. Our data associated these phenotypes with three aberrant DNA methylation signatures: the *LLT1*, *PD*-*L1*, and *Bcl*-*w* hypomethylations. Strikingly, we observed that these three concomitant hypomethylations were only observed in GBM patients having a potential exposure to diuron via their professional activity.

**Conclusions:**

As single player, diuron is not an oncogenic of glioma, but it can participate to the glioma formation in association with other events (also devoid of oncogenic property as single player) such as Akt overexpression.

## Background

The European Cancer Observatory (ECO) estimates the number of brain tumor at 30715 in Europe (http://eco.iarc.fr) and the Central Brain Tumor Registry of the United States (CBTRUS) estimates at 26070 the number of brain primary malignant tumor in 2017 (http://www.cbtrus.org). In 2016, the World health Organization (WHO) classification of central nervous system (CNS) tumors was revised to integrate molecular signatures to histological parameters in order to enhance the robustness of the classification of CNS tumors.

During the last decades, different molecular oncogenic events have been described to induce primary tumor brain formation. Thus, oncogenic overexpression and induction of global DNA hypomethylation are described to promote the gliomagenesis [1–4]. In addition, several risk factors have been described as potential contributors/inducers of glioma risk. These environmental risk factors include allergies, exposure to ionizing and non-ionizing (cellular phones) radiations, exposure to chemicals, solvents, and pesticides [5–7].

A study performed in southwestern of France reports that a high level of occupational exposure to pesticides might be associated with an excess risk of brain tumors [8]. Meta-analysis of brain cancer and farming indicated that pesticides exposure commonly experienced by farmers may contribute to the increased risk of brain cancer [9]. A study performed in Nebraska significantly associated some specific agricultural pesticide exposures and the risk of glioma among male farmers [10]. However, these studies need to be counterbalanced by study reporting the absence of association between pesticide exposure and risk of glioma [7].

Diuron is a herbicide and an antifoulant listed as a likely human carcinogen by US Environmental Protection Agency (USEPA) in 1997. Due to this dual and extensive utilization, diuron contamination is frequently observed [11]. Diuron and its metabolites appear as the third pesticide detected in surface water in Italy with a concentration of 3.10^4^ ng/L [12]. Diuron is detected in Great Barrier Reef lagoon at a concentration exceeding the Australian and New Zealand guideline trigger value of 0.2 μg/L [13]. The CEREMA study reports that diuron is detected in air with a peak at 0.31 ng/m^3^. This study also indicated that diuron is detected in approximately 30% of groundwater samples with a maximum concentration of 0.279 μg/L. The CEREMA study also reports that diuron was detected in 70% of samples with a maximum concentration of 0.864 μg/L and the mean concentration of 0.041 μg/L in European river waters. Diuron is also detected in food and vegetables. European Food Safety Authority mentions an acceptable daily intake (ADI) of 0.007 mg/kg bw/day and an acute reference dose (ARfD) of 0.016 mg/kg bw/day. In a document edited in 2011, EFSA reports that the highest chronic exposure represented 1.8% of the ADI (German child) and the highest acute exposure amounted to 6.1% of the ARfD (apple). Thus, in addition to professionals using diuron in their activity, diuron exposure is multiple for human.

Despite its classification as a likely human carcinogen, little is known about the combination effect of oncogenic overexpression and the pesticide exposure on the glioma occurrence. More generally, little is known about the diuron-induced tumor molecular mechanism even if diuron has been already reported to promote the bladder [14, 15], urothelial [16], skin [17, 18], and mammary [15–19] carcinogenesis.

In this article, we asked the question to determine whether the diuron exposure of glial cells could promote glioma formation and if that is the case, we will analyze the putative involvement of DNA methylation modifications in the diuron-induced glioma.

Here, we show that the unique diuron exposure is not oncogenic for glial cells. However, the combination of diuron exposure and Akt overexpression promote glioma. In terms of molecular mechanisms, our data indicate that the diuron/Akt-induced glioma is characterized by active and passive processes of DNA demethylation that promote an epigenetic reprogramming of certain apoptosis and immune system actors. Thus, this supports the escape of cell death and immune system.

## Methods

### Cell culture and diuron treatment

Ntv-a cell line used was obtained from Holland [1] team and corresponds to newborn tv-a transgenic mice brain cells infected with viruses containing RCAS vectors with LacZ, Ras, and/or Akt. These cells are cultivated with DMEM with 10% fetal calf serum, 1% glutamine, 1% penicillin/streptomycin, and maintained at 37 °C in a humidified atmosphere containing 5% CO_2_-air. Cells were treated with 100 μM of diuron dissolved in DMSO each 2 days during 14 days, control cells received DMSO only.

### Tumorigenicity assay

After diuron exposure, Ntv-a cells were harvested by trypsinization, washed, and resuspended in saline buffer. Cell suspensions were injected s.c. (subcutaneous) as 2.10^6^ cells in 0.05 ml of PBS with equal volume of matrigel matrix (Becton Dickinson, France) in the flank of seven groups of five 7/8-week-old nude NMRI-nu female mice (Janvier, France). Tumor volumes based on caliper measurements were calculated by the modified ellipsoidal formula (tumor volume = 1/2 (length × width^2^)).

The experimental procedures using animals were in accordance with the guidelines of Institutional Animal Care and the French National Committee of Ethics. In addition, all experiments were conducted according to the Regulations for Animal Experimentation at the “Plate-forme Animalerie” of “Institut de Recherche en Santé de l’Université de Nantes (IRS-UN)” and approved by the French National Committee of Ethics (Agreement number: B44278).

### Measure of global DNA methylation

DNA was extracted by using the QiaAmp DNA mini Kit (Qiagen, France). Next, global DNA methylation was estimated by quantifying the presence of 5-methylcytosine using 5-mC DNA ELISA kit (Zymo Research, France) according to the manufacturer’s instructions.

### RT-qPCR analysis

RNA extract is performed using RNeasy Mini QIAcube Kit and QIAcube (Qiagen, France). RT-qPCRs are performed using QuantiTect Reverse Transcription Kit, Rotor-Gene SYBR Green PCR Kit, QuantiTect Primer Assays, and Rotor-Gene Q as real-time thermocycler (Qiagen, France). Reference gene RPLP0 was used, with the 2^-∆∆Ct^ relative quantification method.

### Proximity ligation in situ assay

Cells were cultured for 24 h on cover slip. Cells were then fixed with 4% paraformaldehyde in PBS pH 7.4 for 15 min at room temperature. Permeabilization is performed with PBS containing 0.5% Triton X-100 for 20 min at room temperature. Blocking, staining, hybridization, ligation, amplification, and detection steps were realized according to manufacturer’s instructions (Olink Bioscience, Sweden). All incubations were performed in a humidity chamber. Amplification and detection steps were performed in dark room. Fluorescence was visualized by using the Axiovert 200 M microscopy system (Zeiss, Le Pecq, France) with ApoTome module (X63 and numerial aperture 1.4). Preparations were mounted by using ProLong® Gold antifade reagent with DAPI (Life Technologies, France). Pictures acquisition was realized in structured illumination microscopy. After decovolving (3.5 Huygens Essential software (SVI)), 3D view was obtained by using Amira.4.1.1 program. Finally, images were analyzed by using the freeware “BlobFinder” available for download from www.cb.uu.se/~amin/BlobFinder. Thus, we obtained either number of signals per nuclei since nuclei can be automatically identified. DNMT1 and UHRF1 were detected with anti-DNMT1 (Santa Cruz, sc10221, France) and anti-UHRF1 (Santa Cruz, sc98817, France).

### Measure of genes methylation by quantitative methylation-sensitive restriction enzyme digestion

Quantitative methylation-sensitive restriction enzyme digestion (qMSRE) combines the use of methylation-sensitive restriction enzyme and real-time PCR.

For qMSRE, 500 ng of purified genomic DNA (QIAamp DNA Mini QIAcube Kit and QIAcube (Qiagen, France)) is subject to digestion with adequate methylation sensitive enzymes. For each assay, these enzymes can be changed, but generally, we used enzymes that can digest unmethylated CG sites but not methylated CG sites. A parallel “mock” reaction containing all reaction components except enzyme (replaced with glycerol) is included for each sample. DNA from the digested or mock reaction is then amplified by real-time PCR with specific primers. Thus, 5 μl of digested or mock solutions were used to perform qPCR using the Rotor-Gene SYBR Green PCR Kit and Rotor-Gene Q as real-time thermocycler (Qiagen, France). DNA was digested by AciI, BspEI, and HpaII (4 h at 37 °C) for the *Bcl*-*w*-qMSRE, AciI, and BsrBI (two digestions: 4 h at 37 °C and 4 h at 65 °C) for the *LLT1*-qMSRE or by Hpa I, HhaI, and AvaI (4 h at 37 °C) for the *PD*-*L1*-qMSRE. qPCR was performed with the and the following primers: Bcl-w: CTCTGCTTTTCCTAGGCACGCAA and AGGGCTGTTCAGAGGCCATAGT, LLT1: primers: ACCATCTGGCCTGGATCACA and GGAGAGTTACCCATTTGGCCCATT, and PD-L1: AAATGCAGTGATGGCCCATTTC and GATCCACATAGGTTGCCTTCCTCT. The methylation level for any amplified region can be determined using the following equation Percent Methylation = 100 × 2^-ΔCt^ where ΔCt = the average Ct value from the digested reaction minus the average Ct values from the reference/undigested reaction.

Sequences of the considered promoters were obtained from the Eucaryotic Promoter Database (https://epd.vital-it.ch).

### Measure of cell death

Percentages of cell death were evaluated by using a Trypan Blue Stain 0.4%, and the Countess® Automated Cell Counter (ThermoFisher, France). Cell death was induced using temozolomide (25 μM, Torcis, France). Two Gy irradiation was performed by using X-Rad 225cx (Precision X-Ray inc., North Bradford, CT, USA).

### siRNA transfection protocol

In a six-well culture plate, 2.10^5^ cells were incubated for 24 h at 37 °C in a CO_2_ incubator. Then, 60 pmol of siRNA was added and the cells incubated for 7 h at 37 °C in a CO_2_ incubator. Without removing the siRNA, 1 ml of normal growth medium containing two times the normal serum and antibiotics concentration was added and cells incubated for 24 h. Then, cells were cultured for 48 h in normal culture medium. Thus, analyses were realized 72 h after the siRNA transfection. The following siRNA were used: siRNA-A (control) (sc-37007, Santa Cruz), siRNA-Bcl-w (sc-37294, Santa Cruz) and siRNA-APOBEC3 (sc-60091, Santa Cruz).

#### Measure of Akt and APOBEC3γ expression

The Akt and APOBEC3γ expression level were estimated by ELISA method and the use of the PathScan® Total Akt1 Sandwich ELISA Kit (Cell Signaling, France) and APOBEC3γ ELISA kit (MyBioSource, USA) according to the manufacturer’s instructions.

#### Patient samples

Patient samples were collected from GBM patients treated at the “Institut de Cancérologie de l’Ouest” (ICO, http://www.ico-cancer.fr). All patients recruited gave signed, informed consent. All the samples collected and the associated clinical information were registered in the database (N° DC-2018-3321) validated by the French research ministry. Biological resources were stored at the “Centre de Ressources Biologiques-Tumorothèque” (Institut de Cancérologie de l’Ouest, Saint-Herblain, F44800, France).

### Statistical analysis

All experiments were done at least in triplicates. Significance of the differences in means ± standard deviations was calculated using Student *t* test. Significance of correlation between two parameters was calculated using Pearson’s test.

## Results

### The combination of diuron exposure with Akt overexpression induces glioma, while neither diuron nor Akt alone is sufficient to induce glioma formation

The RCAS/tv-a model has been a very useful and productive tool for studying the gliomagenesis [20]. In this model, PDGF-B overexpression promotes oligodendrogliomas and oligoastrocytomas from neural progenitors and astrocytes, and the combination of activated Ras and Akt induces high-grade gliomas [1], while neither activated Ras nor Akt alone is sufficient to induce GBM formation [2].

**Fig. 1:**
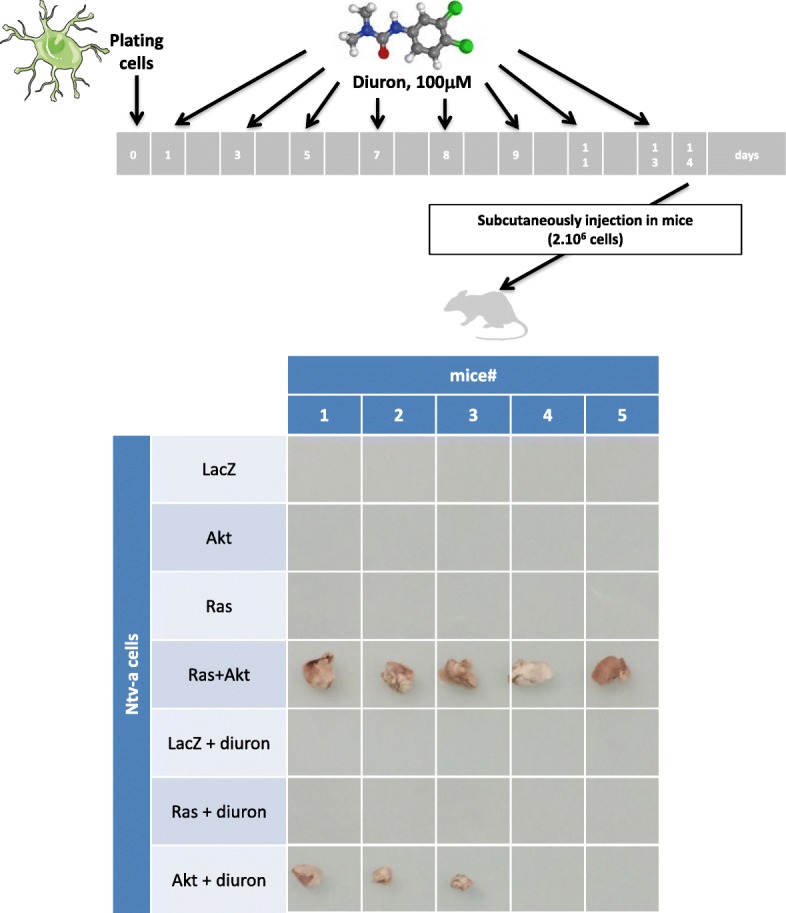
Diuron exposure is not sufficient to induce gliomagenesis, but its combination with Akt overexpression promotes gliomagenesis. Experimental procedures were here summarized. Five mice per condition were xenografted subcutaneously with indicated cells. Sixty days after cells injection, mice were euthanized and tumors were collected. Pictures illustrate the resected tumors

We first have asked the question to know whether the diuron exposure on Ntv-A cells overexpressing LacZ, Ras, or Akt had the ability to promote the gliomagenesis such as the Ras + Akt combination. For this purpose, Ntv-a/LacZ, Ntv-a/Akt, and Ntv-a/Ras cells were exposed to 100 μM diuron each 2 days during 14 days (Fig. 1) to generate Ntv-a/LacZ + diuron, Ntv-a/Akt + diuron, and Ntv-a/Ras + diuron cells. Five independent exposures were performed for each cell types. The diuron exposure dose (100 μM or 23 mg/L) was determined as being (i) a dose devoid of cytotoxicity (Additional file 1: Figure S1), and (ii) a dose inferior to the one seen in human blood (that is 100 mg/L [21]). Tumorigenicity assays were performed via the injection of diuron-exposed cells. Five mice were used for the Ntv-a/LacZ, Ntv-a/Akt, Ntv-a/Ras, and Ntv-a/Ras + Akt cells. Each independent diuron exposure was subcutaneously injected in one mice. As expected, our experiments confirmed that the Ras+ Akt combination acts as oncogenic event for the glioma formation, whereas neither Ras nor Akt alone is sufficient to induce GBM formation (Fig. 1). We next noted that diuron exposure is not sufficient to induce glioma formation, while its combination with Akt promotes the glioma formation in 60% of our experiments (3/5).

**Fig. 2:**
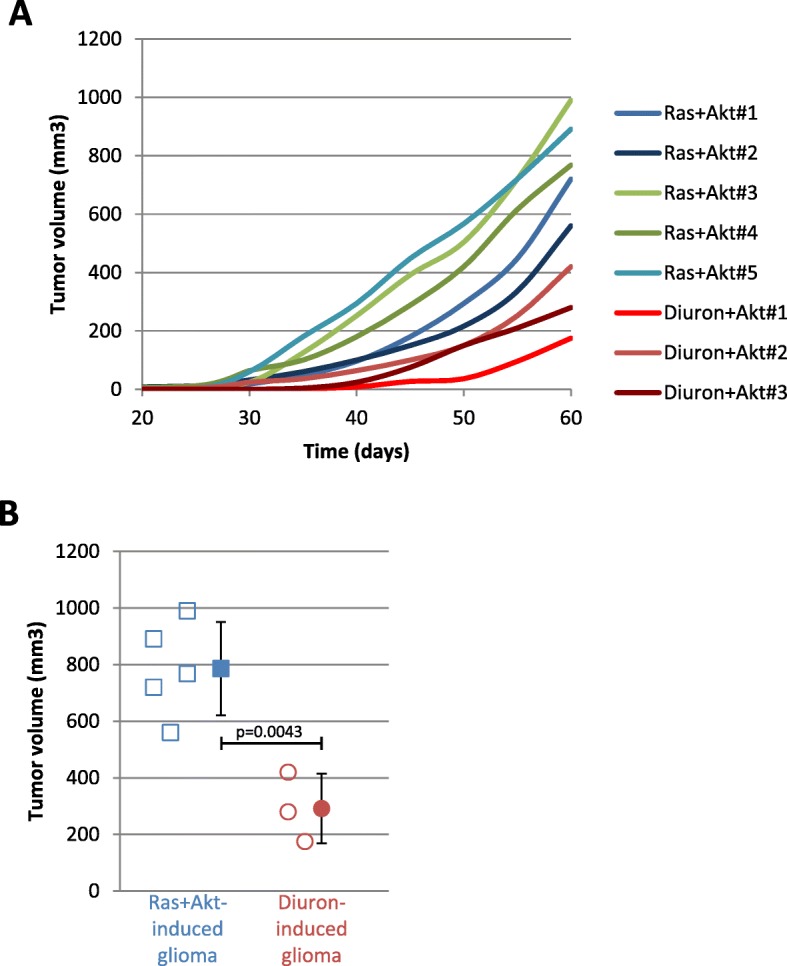
The growth of gliomas induced by the Akt + diuron condition is less aggressive than the one induced by Ras + Akt overexpression. **a** Curves illustrate, for each mice, the tumor growth of Ras + Akt- and Akt + diuron-induced glioma. **b** Graph illustrates and compares, 60 days after the cells injection, the volume of all Ras + Akt-induced glioma (*n* = 5, blue squares) with the volume of all Akt + diuron-induced glioma (*n* = 3, red circles)

Next, we have compared the tumor-forming potential of Ras + Akt cells and Akt + diuron cells by comparing the tumor growth monitored by caliper measurements. Kinetic of tumor growth and the measure of tumor volume indicated that the volume of tumors induced by the Ras + Akt combination and the tumor growth of these tumors were superior than the one induced by the Akt + diuron combination (Fig. 2a, b).

### Cells promoting Akt + diuron-induced tumors are globally hypomethylated via active and passive DNA demethylation processes

**Fig. 3:**
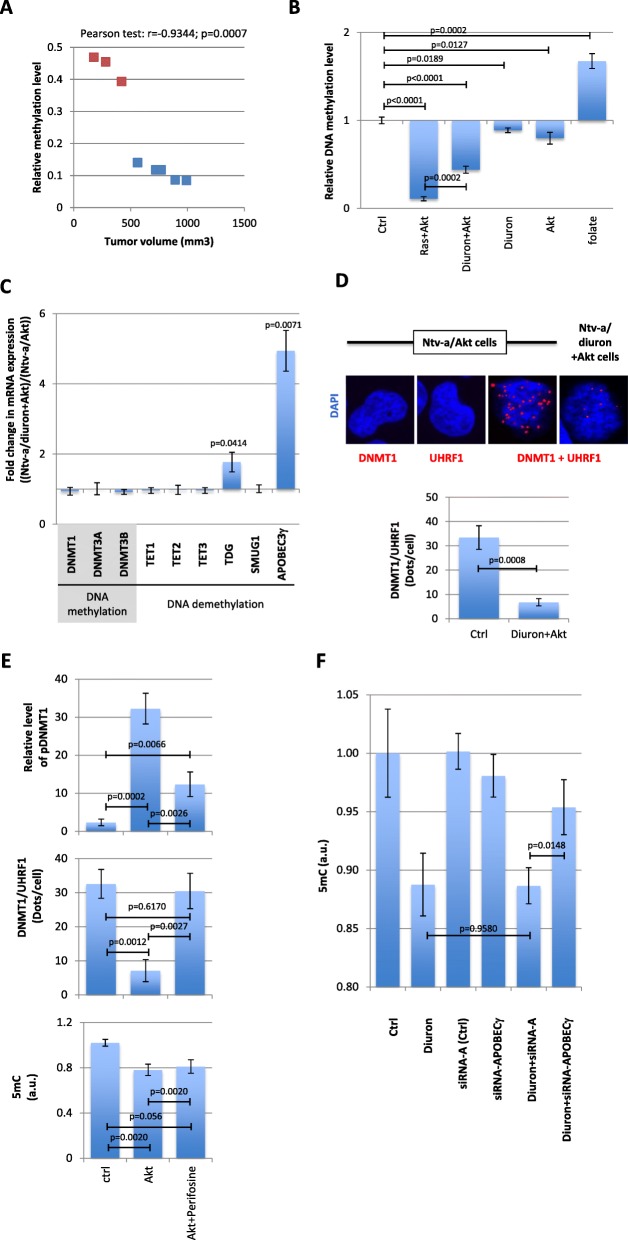
Global DNA hypomethylation occurs in Akt + diuron-induced glioma. **a** Correlation between the relative level of DNA methylation and the tumor volume. Blue squares represent the Ras + Akt-induced glioma (*n* = 5) and red squares represent the Akt + diuron-induced glioma (*n* = 3). **b** Graph illustrates the relative DNA methylation level (mean ± SD) seen in Ntv-a/Ras + Akt- and Ntv-a/diuron + Ak cells (*n* = 3). “Folate” represents the relative DNA methylation level in cells treated with folate, i.e., as a positive control of gain of DNA methylation. “Ctrl” represented the methylation level of Ntv-a/lacZ cells. **c** Graph represents the fold change expression in mRNA encoding for DNA methylation players (DNMT1, 3A, and 3B) and DNA demethylation players (TET1, 2, and 3, TDG, SMUG1, and APOBEC3γ). **d** ApoTome view of the DNMT1/UHRF1 dots in indicated cells. Red dots symbolize the DNMT1/UHRF1 interactions. Nucleus/DNA are stained in blue via the use of DAPI. Graph (mean ± SEM) illustrating the number of DNMT1/UHRF1 dots per cells in indicated cells. The number of DNMT1/UHRF1 dots is calculated from the analysis of, at least, 50 nucleus in three independent experimentations. The absence of dots in the presence of the unique use of DNMT1 or UHRF1 antibodies underlines the specificity of signal obtained in presence of the DNMT1 + UHRF1 antibodies. **e** Graphs illustrate the effect of Akt overexpression and/or inhibition (via the use of Perifosine) on the relative level of phospho-DNMT1 (pDNMT1) (top), the DNMT1/UHRF1 interactions (middle), and on the global level of 5-methylcytosine (5mC, bottom). P-LISA estimates the number of DNMT1/UHRF1 interactions. In cell ELISA performed with anti-pDNMT1^S127^ was used to analyze the DNMT1 phosphorylation such as previously described [3]. ELISA-5mC (Zymo Research, France) was used to quantify the 5methylcytosine (5mC), i.e., to study the global level of DNA methylation. **f** Graph illustrates the modification of 5-methylcystosine seen in cells treated with diuron and/or siRNA directed against APOBEC3γ. ELISA-5mC (Zymo Research, France) was used to quantify the 5methylcytosine (5mC), i.e., to study the global level of DNA methylation

In human glioma, the global level of DNA methylation is associated with the tumor grade and with the aggressiveness of tumors [3, 22, 23]. Our results correlated the relative methylation level of Ras + Akt-induced and Akt + diuron-induced tumors with the tumor volume (Fig. 3a).

To characterize the 5-methylcystosine (5mC) methylation level in cells at the origin of the Akt + diuron-induced glioma (Ntv-a/Akt + diuron cells), we next compared the methylation level of DNA extracted from Ntv-a/LacZ (control, Ctrl), Ntv-a/Ras + Akt, and Ntv-a/Ras + Akt + folate cells. Folate treatment of Ntv-a/Ras + Akt cells (40 μg/ml, 72 h) was here used as a treatment to induced a gain of 5mC. 5mC ELISA reveals that Ntv-a/Akt + diuron cells were hypomethylation in comparison with Ntv-a/LacZ cells (control cells) (Fig. 3b). In addition, we noted that Ntv-a/Ras + Akt cells were more hypomethylated than Ntv-a/Akt + diuron. We also noted that Akt + diuron induced a strong global DNA hypomethylation resulting from synergic effect between the Akt overexpression and the diuron exposure since Akt + diuron DNA demethylation percentage is higher than the theoric addition of diuron effect only and Akt overexpression alone (Fig. 3b).

In order to determine a molecular cause at the origin of the global DNA hypomethylation seen in Ntv-a/Akt + diuron cells, we first analyzed the expression level of several actors of the DNA methylation and DNA demethylation machineries. RT-qPCR analyses indicated that *APOBEC3****γ*** and *TDG* were overexpressed in Ntv-a/Akt + diuron cells compared to Ntv-a/Akt cells (Fig. 3c).

In addition, we also studied the integrity of the DNMT1/UHRF1/PCNA complex through the integrity of the DNMT1/UHRF1 interaction. This focus is due to the fact that the loss of DNMT1/UHRF1/PCNA integrity promotes global DNA hypomethylation and induces tumorigenesis [3, 4]. Our study reports that the DNMT1/UHRF1 signals decrease in Ntv-a/Akt + diuron cells in comparison with Ntv-a/Akt cells (Fig. 3d). Thus, we conclude that the loss of DNMT1/UHRF1/PCNA and the APOBEC3**γ** overexpression are the two leading molecular causes of the global DNA hypomethylation seen in cells at the origin of Akt + diuron-induced glioma.

The above data support the hypothesis that Akt induces the global DNA hypomethylation through the disruption of the DNMT1/PCNA/UHRF1 complex via the DNMT1 phosphorylation, such as previously described [3]. To address this point, we analyzed the impact of an Akt inhibitor on the level of pDNMT1, the level of DNMT1/UHRF1 interactions, and 5mC. Our data confirmed that Akt overexpression promotes the DNMT1 phosphorylation, the decrease of DNMT1/UHRF1 interaction, and the decrease of 5mC in DNA studied (Fig. 3e). We also observed that Perifosine, an Akt inhibitor, limited the DNMT1 phosphorylation and restored the DNMT1/UHRF1 interactions but is ineffective to restore the global level of DNA methylation (Fig. 3e).

Concerning the diuron-mediated global DNA hypomethylation, our data suggest a mechanism mainly involving the APOBEC3γ overexpression. To investigate this point, we analyzed the impact of a siRNA-mediated APOBEC3γ downregulation on the global level of 5mC. Our results indicated that siRNA-APOBEC3γ abrogated the diuron-mediated global DNA hypomethylation, while siRNA-A (control) has no effect on the diuron-mediated global DNA hypomethylation (Fig. 3f).

### Cells promoting Akt + diuron-induced gliomas are resistant to the temozolomide/irradiation-induced cell death through the Bcl-w overexpression

**Fig. 4:**
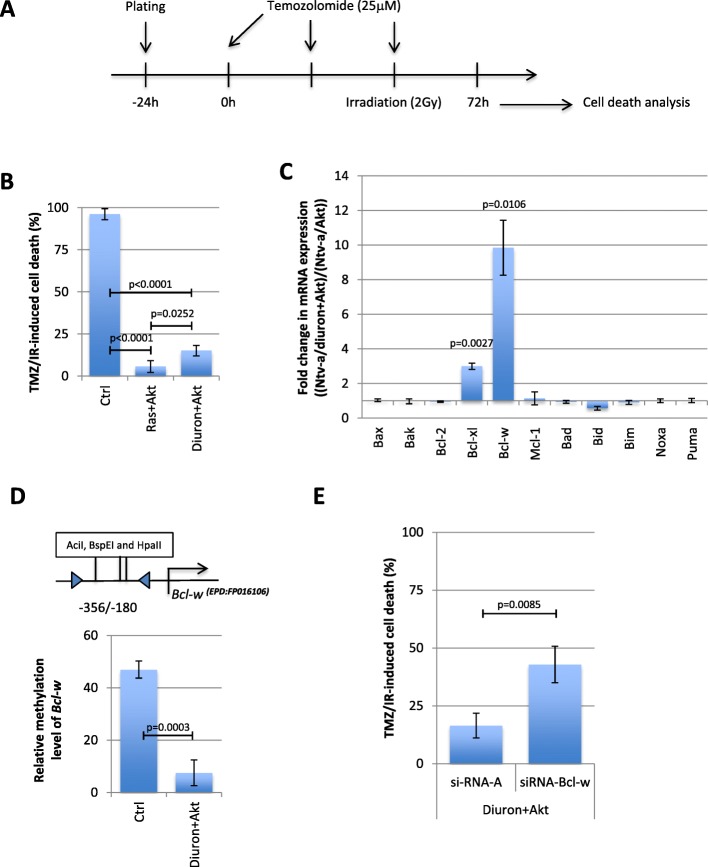
The Akt + diuron-induced gliomas are resistant to the temozolomide/irradiation-induced cell death. **a** Schematic representation of the temozolomide/irradiation-induced cell death. **b** Graph compares the percentage of temozolomide/irradiation-induced cell death in indicated cells according to Trypan blue method. **c** Graph represents the fold change expression in mRNA encoding for 11 proteins of the BCL2 family. **d** qMSRE estimates the relative level of DNA methylation of *Bcl*-*w* promoter. **e** Graph compares the percentage of temozolomide/irradiation-induced cell death in indicated cells according to Trypan blue method

The standard anti-GBM therapy includes a surgical resection followed by radiation and chemotherapy using temozolomide. Thus, we first studied whether Ntv-a/Akt + diuron cells are sensitive to the temozolomide/radiation therapy. For this purpose, we have calculated the percentage of cell death in Ntv-a/LacZ (Ctrl), Ntv-a/Ras + Akt, and Ntv-a/Akt + diuron. For this purpose, cells were treated with 25 μM of temozolomide (TMZ) for 72 h and irradiated (2 Gy) (Fig. 4a). Thus, we noted that Ntv-a/Ras + Akt and Ntv-a/Akt + diuron cells were strongly resistant to the temozolomide/radiation-induced cell death since this treatment promoted the cell death in a small percentage of cells (5% and 15%, respectively) (Fig. 4b). In order to determine a molecular cause at the origin of this resistance, we analyzed the expression level of 11 apoptotic players. RT-qPCR experiments indicated that mRNA encoding for two anti-apoptotic proteins were overexpressed in Ntv-a/Akt + diuron cells in comparison with Ntv-a/Akt cells: threefold for *Bcl*-*xl* and near to tenfold for *Bcl*-*w* (Fig. 4c). qMSRE analyzing the methylation level of the *Bcl-w* promoter indicated that the promoter region of *Bcl-w* was hypomethylated in Ntv-a/Akt + diuron cells in comparison with Ntv-a/Akt cells (Fig. 4d).

In order to determine whether the diuron-induced Bcl-w overexpression played a crucial role on the temozolomide/radiation resistance, we then measured the percentage of temozolomide/radiation-induced cell death in Akt + diuron cells presenting a Bcl-w downregulation induced through the use of siRNA directed against Bcl-w (Additional file 1: Figure S2). This experiment indicated that siRNA-Bcl-w induced gain of 26% of TMZ/IR-induced cell death (Fig. 4e). This finding confirms that the Bcl-w overexpression plays a role in the TMZ/IR-resistance phenotype seen in cells treated with Akt + diuron.

### Cells promoting Akt + diuron-induced gliomas are characterized by the DNA hypomethylation-mediated PD-L1 and LLT1 overexpression

**Fig. 5:**
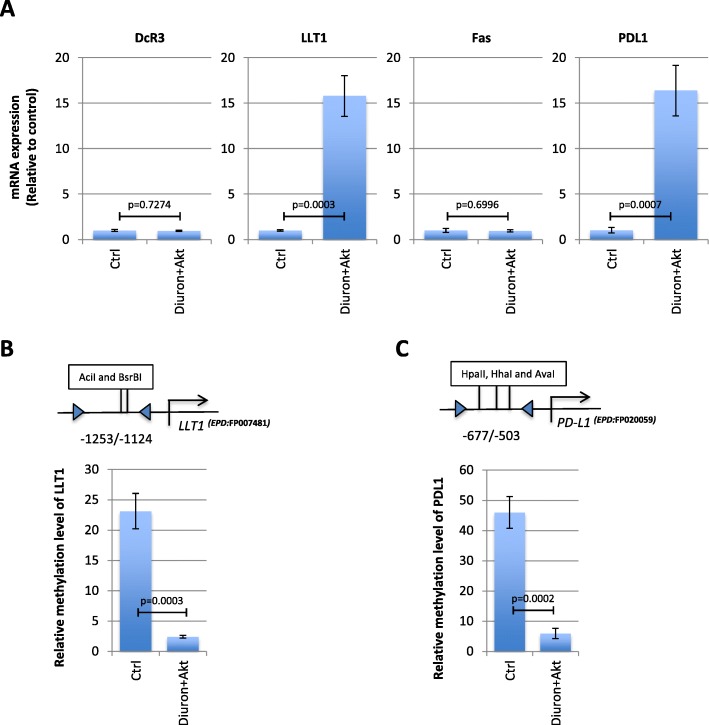
Akt + diuron-induced gliomas are characterized by the DNA hypomethylation-mediated PD-L1 and LLT1 overexpression. **a** Graphs compare the relative mRNA expression encoding for immune players (DcR3, LLT1, and PD-L1 for immunotolerance, and Fas for immunostimulation) in control (Ctrl) and diuron + Akt cells. **b** qMSRE estimates the relative level of DNA methylation of *LLT1* promoter. **c** qMSRE estimates the relative level of DNA methylation of PD-L1 promoter

Among the various mechanisms of resistance acquired by tumor cells during tumorigenesis, there is the immune system evasion. A pleiotropic number of molecular signatures can be associated to the immune escape of glioma. Here, we have decided to focus our study on four main actors of the immune escape of glioma cells: PD-L1 (since PD-L1 is upregulated in glioma [24]), Fas (a crucial player of the immune system-induced apoptosis [25]), DcR3 (a negative regulator of Fas [26]), and LLT1 (since LLT1 acts as a mediator of immune escape [27]). Our data indicated that Akt + diuron cells overexpressed PD-L1 and LLT1 (Fig. 5a). qMSRE analyses indicated that LLT1 and PD-L1 promoter were hypomethylated in Akt + diuron cells (Fig. 5b, c).

### Hypomethylation of *Bcl*-*w* and *PD*-*L1* promoters is associated with the APOBEC3γ overexpression, while hypomethylation of *LLT1* promoter is associated with the DNMT1/PCNA/UHRF1 disruption

**Fig. 6:**
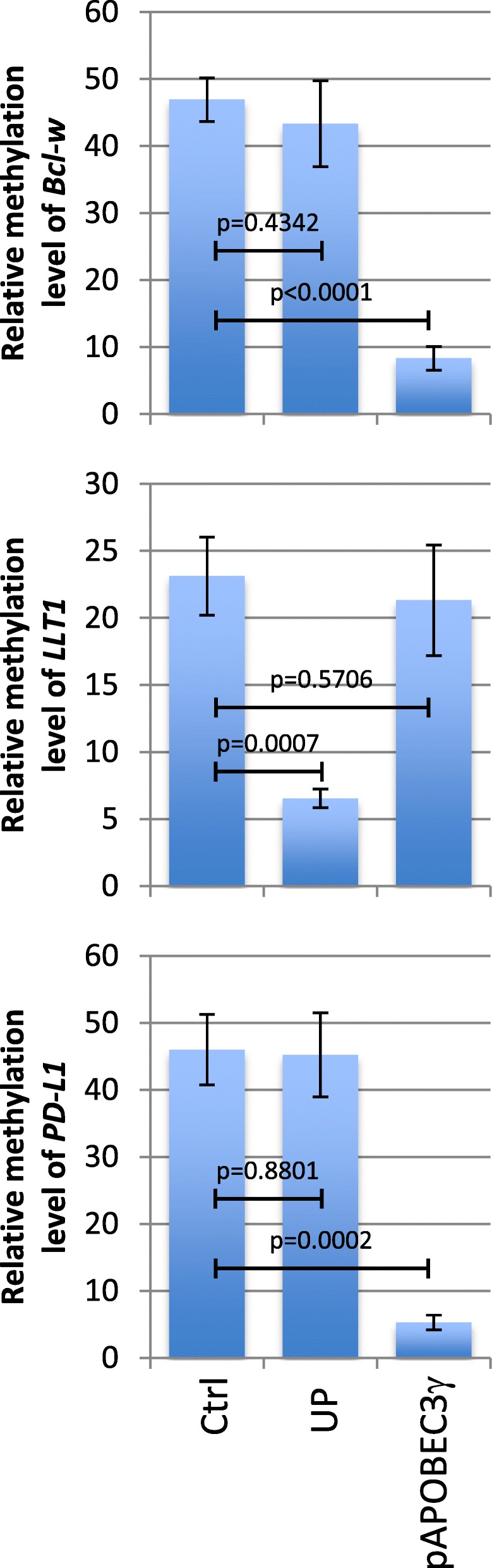
APOBEC3γ promotes the PD-L1 and Bcl-w demethylation, while the DNMT1/PCNA/UHRF1 disruption promotes the LLT1 demethylation. qMSRE (as previously described) compare the relative methylation level of *Bcl*-*w*, *LLT1*, and *PD*-*L1* genes in cells transiently transfected with the pcDNA.3.1 (Ctrl), pcDNA.3.1-UP plasmid, and with pcDNA.3.1-APOBEC3γ plasmids. pcDNA.3.1-UP is a plasmid encoding for a chimeric protein having the ability to selectively disrupt the DNMT1/UHRF1 interaction [3, 4]

Akt + diuron cells presenting an APOBEC3**γ** overexpression and a loss of DNMT1/UHRF1/PCNA, we hypothesized that these two causes of DNA hypomethylation could be involved in the hypomethylation of *Bcl*-*w*, *PD*-*L1*, and *LLT1* promoters. To test this hypothesis, APOBEC3**γ** was overexpressed by transfecting a plasmid coding for this protein (Additional file 1: Figure S3), while the loss of DNMT1/UHRF1/PCNA complex was obtained through the use of a plasmid encoding for a peptide disrupting this complex (named UP, such as previously described [3, 4]). qMSRE analyses indicated that the APOBEC3**γ** overexpression promoted the *Bcl*-*w* and *PD*-*L1* hypomethylation, while the UP-induced loss of DNMT1/UHRF1/PCNA complex induced the LLT1 hypomethylation (Fig. 6).

### Presence of concomitant *LLT1*, *PD*-*L1*, and *Bcl*-*w* hypomethylation in GBM patients

**Fig. 7:**
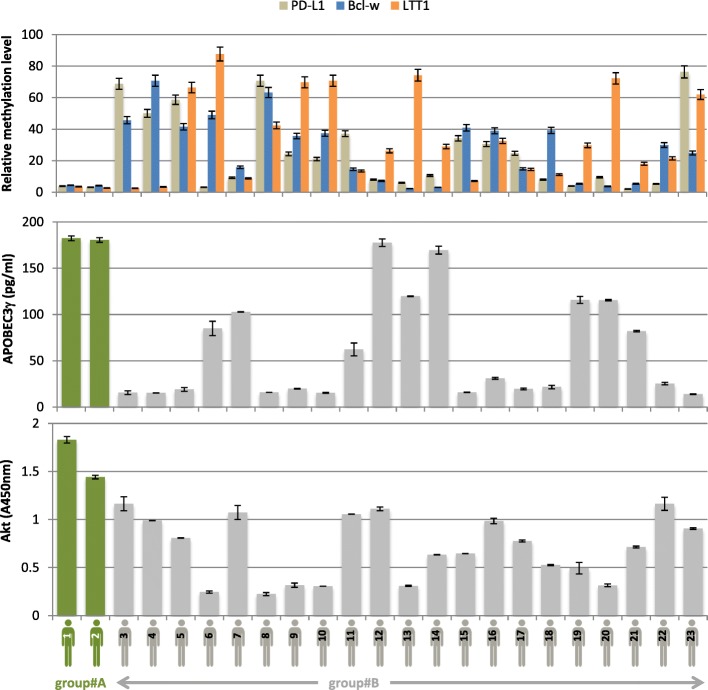
Methylation status of *PD*-*L1*, *Bcl*-*w*, and *LLT1* genes in GBM patients putatively exposed to diuron via their professional activity (green, *n* = 2) or not (gray, *n* = 21) qMSRE (as previously described) compare the relative methylation level of *Bcl*-*w*, *LLT1*, and *PD-L1* genes. APOBEC3γ and AKT expressions were estimated by ELISA method. GBM patients potentially exposed to diuron via their professional activity (farmer, gardener …) are in green (*n* = 2), and GBM patients potentially unexposed to diuron via their professional activity are in gray (*n* = 21).

Our data indicate that the gliomagenesis induced by the Akt overexpression and diuron exposure is associated with the hypomethylation of the *LLT1*, *PD*-*L1*, and *Bcl*-*w* genes. In other terms, our data indicate that the *LLT1*, *PD*-*L1*, and *Bcl*-*w* hypomethylation are associated with the diuron-induced modifications of methylome. Based on this finding, we hypothesized that GBM patients having had a potential exposure to diuron via their professional activity could have tumors characterized by the concomitant *LLT1*, *PD*-*L1*, and *Bcl*-*w* hypomethylations. To investigate this idea, a GBM sample cohort of 23 patients was used (Additional file 2: Table S1). Interestingly, two of these patients have had a potential exposure to diuron via their professional activity (group#A), while all other patients have a supposed absence of diuron contact (group#B). qMSRE indicated that the concomitant *LLT1*, *PD*-*L1*, and *Bcl*-*w* hypomethylation (defined by a relative methylation level inferior to 5) was only observed in group#A and not in group#B (Fig. 7). We also noted that the two GBM patients included in group#A have high levels of APOBEC3γ and Akt expressions (Fig. 7).

## Discussion

During the last decades, several works define the gliomagenesis as a tumor development generated by multistep and/or chromothripsis (a phenomenon characterized by thousands of chromosomal rearrangements) processes that involve various genetic and/or epigenetic alterations. Thus, during tumor initiation step, genetic and/or epigenetic alterations can promote the acquisition of cancer hallmarks such as the apoptosis and immune evasion. In previous articles, we and others have begun to unveil the mechanisms through which DNA hypomethylation or the Akt/Ras overexpression can promote gliomagenesis and the acquisition of apoptosis and immune evasion phenotypes [3, 4, 28, 29]. However, in these papers, DNA hypomethylation was induced by a chimeric peptide having the ability to disrupt the integrity of the main complex responsible for the DNA inheritance, the DNMT1/PCNA/UHRF1 complex.

In the present article, we report that Akt overexpression and diuron exposure promote the glioma formation from neural progenitor cells via the induction of DNA hypomethylation mediated by two distinct pathways: the loss of DNMT1/PCNA/UHRF1 interactions and the APOBEC3γ overexpression. The identification of these two molecular causes of hypomethylation reinforce the idea that DNMT1/PCNA/UHRF1 play a crucial role in the maintenance of DNA methylation level, such as already described in several articles [3, 4, 30, 31]. This last point provides a new evidence for the involvement of APOBEC3γ in DNA demethylation mechanisms [32, 33], despite the discussion surrounding the role of APOBEC3γ on these mechanisms (a discussion mainly associated with the publication of Wijesinghe and Bhagwat [34]).

Our work is the first to report the evidence that the diuron exposure can promote to the gliomagenesis. However, in gliomagenesis, the oncogenic effect is not due to itself since diuron needs to be associated to Akt overexpression to promote the gliomagenesis. The involvement of diuron in carcinogenesis is not new since diuron is already reported for the bladder [14, 15], urothelial [16], skin [17, 18], and mammary [15–19] carcinogenesis. Nevertheless, none of these publications mentioned an impact of diuron on the global level of DNA methylation and on the methylation level of certain gene promoters.

The fact that diuron needs to be associated with another oncogenic hit (Akt overexpression) to promote gliomagenesis reinforces the idea that tumorigenesis can result from the accumulation of several hits such as mentioned in the Knudson hypothesis, also known as the two-hit hypothesis [35, 36] .

To our knowledge, the impact of diuron on methylome is to date only reported in oyster [37–39]. More generally, several publications report the fact that pesticide exposure affects the methylome. Recently, Rusiecki et al. [40] reports that pesticide exposure has been associated with acute and chronic adverse health effects and that DNA methylation may mediate these effects. Besides in this article, the authors report that pesticide exposure could modulate the *MGMT* methylation. In our study, we noted that the methylation of *MGMT* methylation was unchanged (Additional file 1: Figure S4). We also observed that the two GBM patients potentially exposed to diuron via their professional activity are “MGMT unmethylated.” Zhang et al. [41] report that diazinon-treated cells exhibited increased DNA methylation levels in certain genes defined as tumor suppressor genes such as RASSF1A and PTEN. A finding supporting the idea that the diazinon-induced hypermethylated could play a pathological role in cancer development [41]. On contrary to this study, our work shows that the Akt + diuron-induced tumors are characterized by both global and local DNA hypomethylations. Our data clearly indicated that the diuron + Akt-induced tumorigenesis is associated with the hypomethylation and the overexpression of genes that responsible for apoptosis (Bcl-w) and immune evasion (PD-L1 and LLT1). Indeed, *bcl*-*w*/*bcl2l2* (bcl-2-like protein 2) gene encodes an anti-apoptotic protein which expression is regulated by DNA methylation in glioma [42, 43]. In addition, Bcl-w expression contributed to the aggressiveness of glioma [44]. Lectin-like transcript-1 (LLT1) is a newly identified ligand for the inhibitory natural killer (NK) cell receptor CD161 which the expression acts as a mediator of immune escape and contributes to the immunosuppressive properties of glioma cells [27]. PD-L1 is a protein encoded by the CD274 gene, which plays a major role in suppressing the immune response. The high PD-L1 expression is associated with a worse outcome of patients suffering from GBM according to certain study [45], while others studies contradict this finding [46]. Be that as it may on this point, our observation associating the Akt + diuron-induced demethylation/overexpression of Bcl-w, PD-L1,, and LLT1 is consistent with the Akt + diuron-induced gliomagenesis. In other terms, it appears that diuron promotes the reprogramming of expression of certain apoptotic and immune actors. Other pesticides have the same effect. Thus, we recently observed that a pesticides mixture modulates the Mcl1 expression in MSC cells [47].

Finally, we made the dual observing that (1) the gliomagenesis induced by the Akt overexpression and diuron exposure is associated with the hypomethylation of the *LLT1*, *PD*-*L1*, and *Bcl*-*w* genes and (2) the concomitant *Bcl*-*w*, *PD*-*L1*, and *LLT1* hypomethylation occurs in 2/2 tumors of patients having had a potential exposure to diuron via their professional activity. We did not observe this situation in 21 tumors of patients devoid of potential exposure to diuron via their professional activity. These observations are troubling and tend to support the idea that diuron exposure can play a role of one oncogenic hit on the road to gliomagenesis.

## Conclusion

**Fig. 8:**
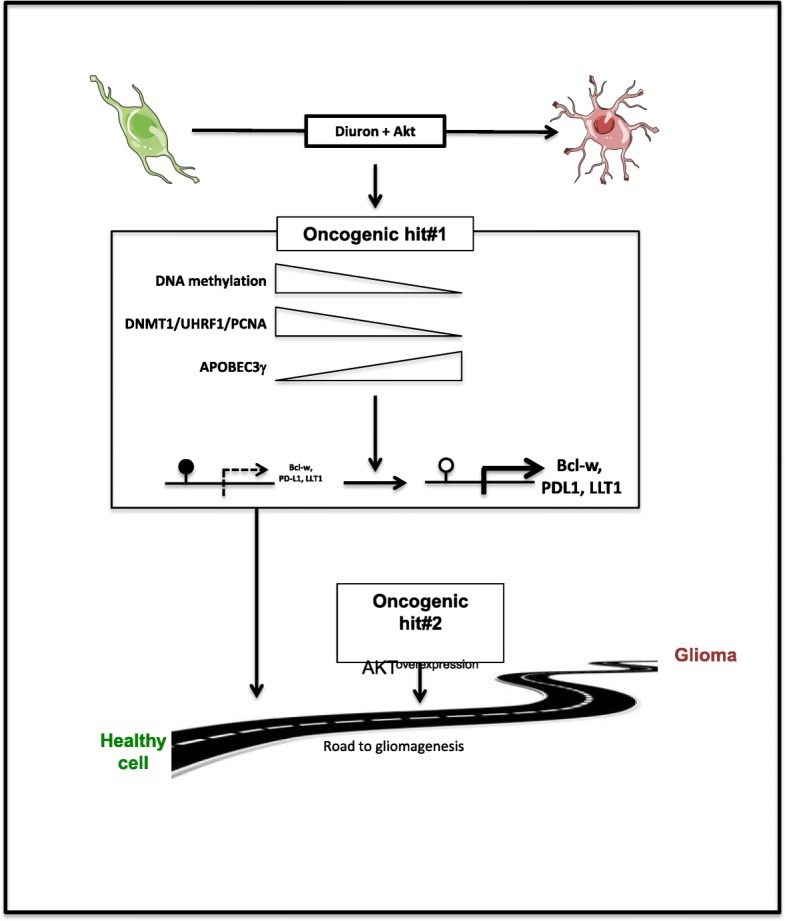
Signaling pathway of the Akt + diuron-induced glioma

Since several years, a large number of studies analyzed the pesticide exposure and the carcinogenesis initiation. Our data strongly identify a causal link between diuron exposure and cancer development. However, our data underline that diuron is not oncogenic by itself since diuron promotes gliomagenesis only when its exposure is associated with the Akt overexpression (another non-oncogenic event of gliomagenesis by itself) in our model. Mechanistically, we determined that diuron promotes gliomagenesis through the induction of DNA methylation modification. Thus, a better understanding of the diuron-induced alterations of DNA methylation mechanisms could provide rational biomarkers for detecting diuron-induced glioma and to design efficient therapeutic strategies against these types of tumors. In addition to our finding, several reports mention the idea that the exposure of environmental chemicals can modulate the epigenome to potentialize or directly induce the carcinogenesis [48–50]. Thus, epigenetic reprogramming by environmental chemical could represent a novel mechanism to explain their carcinogen impact. Thus, without being oncogenic alone, exposure to an environmental chemical such as diuron could be the first step on the carcinogenesis road. And this step needs to “be complete” by other(s) step(s) (such as exposure to other(s) environmental chemical(s) or the genes overexpression, mutation, or deletion, etc...) in order to arrive at its term, i.e., the tumor formation (Fig. 8).

## Supplementary information

**Additional file 1:** **Figure S1.** Cell viability of Ntv-a/lacZ cells exposed to diuron. Different doses of diuron were incubated on Ntv-a/lacZ cells. XTT Cell Viability Kit (Ozyme, France) was used to calculate the percentage of cell viability after 48h of diuron. The values are means±SD from three independent experiments performed in duplicate. **Figures S2 and S3.** RT-qPCR were done to validated the siRNA downexpression. Down-regulation of Bcl-w and APOBEC3γ were performed via cells transient transfection with siRNA-Bcl-w (Santa-Cruz, sc-37294, France) and siRNA-APOBEC3γ (Santa-Cruz, sc-60091, France). siRNA-A is a control (Santa-Cruz, sc-37007, France). **Figure S4.** Graphs illustrate the effect of Diuron+Akt on the MGMTmRNA expression (A) and the MGMT methylation level (B). RT-qPCR estimates the MGMTmRNA expression. qMSRE (OneStep qMethyl™ Kit - Zymo Research, France) estimates the MGMT methylation level

**Additional file 2:** **Table S1.** Characteristics of BGM patients.

## Data Availability

The datasets supporting the conclusions of this article are included within the article and its additional files. All other datasets used and analyzed during the current study are available from the corresponding author on reasonable request.

## Abbreviations

5mC: 5-Methylcystosine
P-LISA: Proximity ligation in situ assay
qMSRE: Quantitative methylation-sensitive restriction enzyme digestion
