# Supplementary figures and images for: Diuron exposure and Akt overexpression promote glioma formation through DNA hypomethylation
Source: Clin Epigenetics. 2019 Nov 14;11:159. doi: 10.1186/s13148-019-0759-1 (PMC6854743; doi:10.1186/s13148-019-0759-1)

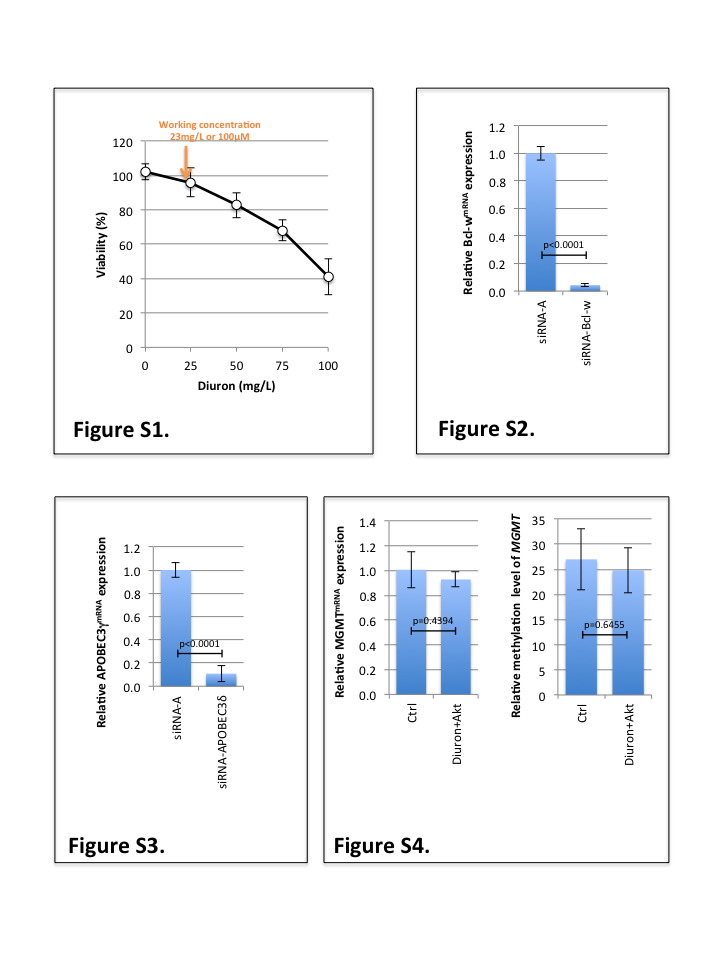

Supplement: Supplementary file 1 — Additional file 1: Figure S1. Cell viability of Ntv-a/lacZ cells exposed to diuron. Different doses of diuron were incubated on Ntv-a/lacZ cells. XTT Cell Viability Kit (Ozyme, France) was used to calculate the percentage of cell viability after 48h of diuron. The values are means±SD from three independent experiments performed in duplicate. Figures S2 and S3. RT-qPCR were done to validated the siRNA downexpression. Down-regulation of Bcl-w and APOBEC3γ were performed via cells transient transfection with siRNA-Bcl-w (Santa-Cruz, sc-37294, France) and siRNA-APOBEC3γ (Santa-Cruz, sc-60091, France). siRNA-A is a control (Santa-Cruz, sc-37007, France). Figure S4. Graphs illustrate the effect of Diuron+Akt on the MGMTmRNA expression (A) and the MGMT methylation level (B). RT-qPCR estimates the MGMTmRNA expression. qMSRE (OneStep qMethyl™ Kit - Zymo Research, France) estimates the MGMT methylation level [file 13148_2019_759_MOESM1_ESM.png]

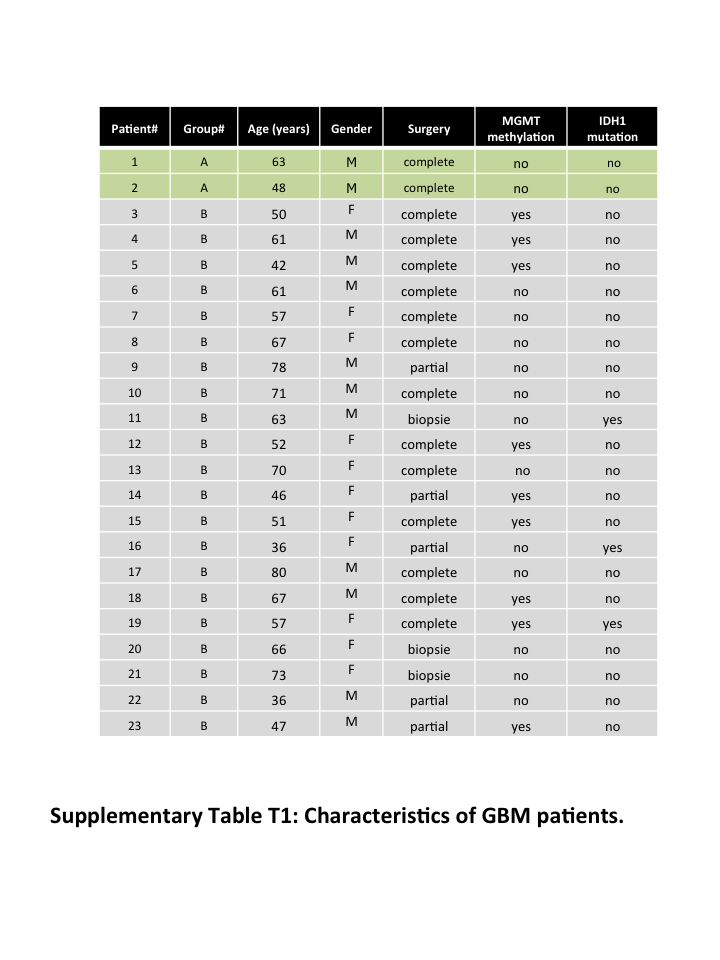

Supplement: Supplementary file 2 — Additional file 2: Table S1. Characteristics of BGM patients. [file 13148_2019_759_MOESM2_ESM.png]
